# Proteomics and Extracellular Vesicles as Novel Biomarker Sources in Peritoneal Dialysis in Children

**DOI:** 10.3390/ijms23105655

**Published:** 2022-05-18

**Authors:** Chiara Trincianti, Vincenzo Meleca, Edoardo La Porta, Maurizio Bruschi, Giovanni Candiano, Andrea Garbarino, Xhuliana Kajana, Alberto Preda, Francesca Lugani, Gian Marco Ghiggeri, Andrea Angeletti, Pasquale Esposito, Enrico Verrina

**Affiliations:** 1 Department of Neuroscience, Rehabilitation, Ophthalmology, Genetics and Mother-Child Health, University of Genoa, 16132 Genoa, Italy; chiara.trincianti@gmail.com (C.T.); vice14@hotmail.it (V.M.); 2 Department of Nephrology, Dialysis and Transplantation, IRCCS Istituto Giannina Gaslini, 16147 Genoa, Italy; edoardo01laporta@gmail.com (E.L.P.); francescalugani@gaslini.org (F.L.); gmarcoghiggeri@gaslini.org (G.M.G.); andreangeletti@gaslini.org (A.A.); 3 Laboratory on Molecular Nephrology, Division of Nephrology, Dialysis, Transplantation, IRCCS Istituto Giannina Gaslini, 16147 Genoa, Italy; maurziobruschi@gaslini.org (M.B.); giovannicandiano@gaslini.org (G.C.); andreagarbarino@gaslini.org (A.G.); xhuliakajana@gmail.com (X.K.); 4 Department of Cardiology, Vita-Salute San Raffaele University, 20019 Milan, Italy; albpreda@gmai.com; 5 Unit of Nephrology, Dialysis and Transplantation, Department of Internal Medicine, University of Genoa and IRCCS Ospedale Policlinico San Martino, 16132 Genoa, Italy; 6 Dialysis Unit, Department of Pediatric, IRCCS Istituto Giannina Gaslini, 16147 Genoa, Italy; enricoverrina@gaslini.org

**Keywords:** pediatric peritoneal dialysis, proteomics, exosomes, extracellular vesicles, mesothelial cells, inflammation

## Abstract

Peritoneal dialysis (PD) represents the dialysis modality of choice for pediatric patients with end-stage kidney disease. Indeed, compared with hemodialysis (HD), it offers many advantages, including more flexibility, reduction of the risk of hospital-acquired infections, preservation of residual kidney function, and a better quality of life. However, despite these positive aspects, PD may be associated with several long-term complications that may impair both patient’s general health and PD adequacy. In this view, chronic inflammation, caused by different factors, has a detrimental impact on the structure and function of the peritoneal membrane, leading to sclerosis and consequent PD failure both in adults and children. Although several studies investigated the complex pathogenic pathways underlying peritoneal membrane alterations, these processes remain still to explore. Understanding these mechanisms may provide novel approaches to improve the clinical outcome of pediatric PD patients through the identification of subjects at high risk of complications and the implementation of personalized interventions. In this review, we discuss the main experimental and clinical experiences exploring the potentiality of the proteomic analysis of peritoneal fluids and extracellular vesicles as a source of novel biomarkers in pediatric peritoneal dialysis.

## 1. Introduction

**Figure 1:**
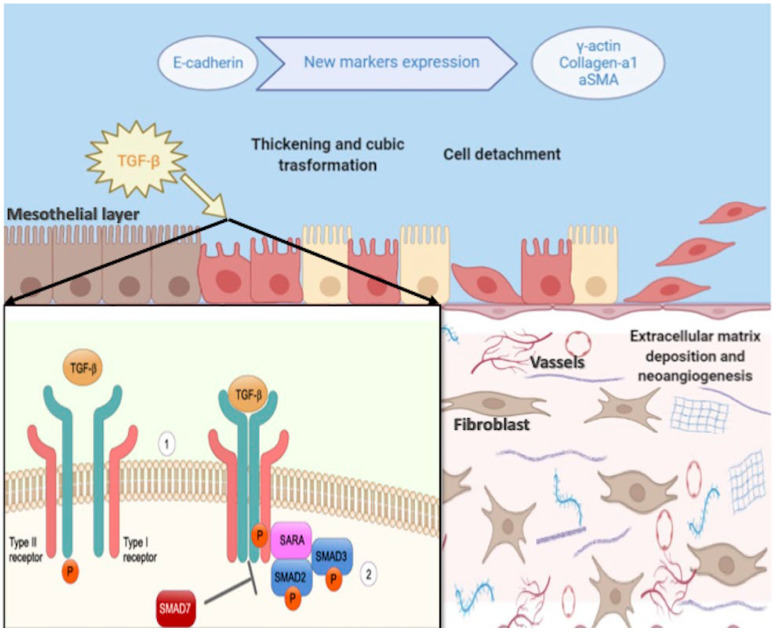
Epithelial to mesenchymal transition of peritoneal membrane. A schematic representation of the peritoneal membrane composed of mesothelial cells monolayer and extracellular matrix within vessels and fibroblast and the progressive change of the structure due to epithelial-to-mesenchymal (EMT) transition. Moreover, the main markers that characterize the phenotype switch of mesothelial cells to fibroblast-like phenotype due to EMT are represented and reported. In the mirror below on the left, the most significant intracellular pathways of the TGF-β signaling are represented: (1) Dimeric TGF-β receptor type I and II before binding the ligand. (2) TGF-β binds receptors and, through phosphorylation, activates SMADs and Shad anchor for receptor activation (SARA) complexes. (TGFβ = transforming growth factor 1-β, αSMA = α smooth muscle actin).

Peritoneal dialysis (PD) represents the preferred dialysis modality for pediatric patients with end-stage kidney disease (ESKD) [1]. Indeed, although transplantation is the gold standard for children with ESKD, many patients need dialysis before transplantation [2]. PD works by taking advantage of the peculiar characteristics of the peritoneal membrane (PM) constituted by a monolayer of mesothelial cells (MC) sustained by a submesothelial zone composed of a matrix containing fibroblasts, collagen, and other extracellular matrix material, at the bottom of which there is a dense network of capillaries [3]. This complex structure is semipermeable and highly vascularized, allowing ultrafiltration and solute removal through the exchanges of a dialysis solution into the peritoneal cavity [4]. The PD solutions consist of physiological concentrations of chloride, calcium, sodium, and magnesium, as well as buffers (lactate and/or bicarbonate) and an osmotic vehicle (glucose is the most used) [5]. Compared with hemodialysis (HD), PD presents some advantages. It may be performed with minimal infrastructural support, is more flexible, minimizes the risk of hospital-acquired infections, and is associated with better preservation of residual kidney function [6]. Moreover, patients on PD reported a better quality of life when compared with HD [7]. These features make PD especially suitable for pediatric patients with ESKD, in whom PD may have the potential to allow a relatively normal life [8]. However, despite these positive aspects, PD is not risk-free. For this reason, apart from early problems linked to surgery and anatomical issues, prolonged therapy with PD may be associated with several long-term complications that may impair both patient’s general health and PD lifespan [9]. In particular, the presence and persistence of inflammatory status may have negative effects on the structure and function of PM, leading to membrane fibrosis and consequent PD failure [10]. The reasons for chronic inflammation in PD may be various, including factors related to the loss of renal function, commonly found in all patients with chronic kidney disease (CKD), such as conditions related to PD treatment itself [11]. Thus, it has been found that the retention of uremic toxins can damage PM through the formation of carbonyl products [12]. Moreover, the occurrence of peritonitis, such as the contact with bioincompatible solutions and the chronic exposure to high glucose concentrations, may be detrimental to PM physiology and MC function [13,14]. PM damaged from inflammation undergoes progressive fibrosis, angiogenesis, and vasculopathy, leading to peritoneal sclerosis [15]. Most importantly, the structural alterations of the PM may have large clinical implications resulting in PD inadequacy and ultrafiltration failure (UFF), which can occur in up to 30% of patients on PD after five years of treatment [16]. Rarely prolonged inflammatory triggers can determine, both in adult and pediatric patients, the development of encapsulating peritoneal sclerosis (EPS), a life-threatening disorder characterized by UFF, intestinal obstruction due to persistent and severe intraabdominal inflammation, encapsulation of the bowels, and malnutrition [17]. Conventional PD solutions are associated with EPS because of acid pH, high osmolality, high lactate concentration, and high-glucose degradation products (GDP) originating from sterilization at high temperatures [18]. Currently, there are no consensus guidelines on EPS therapy: PD discontinuation and immunosuppressive drugs are the gold standards, while surgical intervention is earmarked for patients with severe EPS [19]. The pathogenesis of PM alterations in the course of inflammatory processes is complex and multifaceted, involving both cellular and molecular mechanisms [20,21]. In turn, cellular processes include leukocyte infiltration and generation of myofibroblasts activated by extracellular signals, such as growth factors and cytokines [21]. Among them, transforming growth factor (TGF)-β1 is considered the main responsible for the genesis of peritoneal fibrosis, as increased levels of TGF-beta1 in peritoneal dialysate are associated with worse PD consequences [22]. Moreover, TGF-β1 induces several pro-fibrotic events, including epithelial-mesenchymal transition (EMT), the proliferation of fibroblasts, and the deposition of the matrix [23] (Figure 1). To counteract inflammation-related complications, different PD solutions with neutral pH and low GPD concentrations have been introduced into medical practice [24,25]. Although they modulated immune response and reduced EMT and local inflammation, prospective studies are needed to define their long-term effects [26,27].

In the light of the dynamic conditions of PM and its potential structural and functional changes, there is a need for reliable biomarkers to improve the clinical outcome of PD patients, identifying the ones at high risk of complications and guiding personalized intervention. The research of novel biomarkers is of great interest in the nephrology field since they can increase our knowledge of pathological mechanisms. Their implementation in the clinical practice could improve patient management or outcome prediction accuracy [28].

In the last decades, the characterization of biomarkers and the possibility of extending their use have been explored in different settings of kidney diseases, both in adults and children [29]. This progress was made possible also by the introduction of innovative approaches, such as the -omic methods [30].

A step forward has been represented by the application of proteomics to urinary-derived extracellular vesicles (EVs) and exosomes, which represent a promising source of non-invasive biomarkers. In this regard, PD may be considered an ideal setting to explore these potentialities offered by these new approaches since peritoneal dialysis effluent (PDE) appears as a potential source of biomolecules for clinical application [31] (Table 1). In 2013, Barreto et al. showed that IL-6 levels in PDE could be a feasible biomarker for local peritoneal inflammation, such as the decrease of CA125, a high-molecular-weight glycoprotein that seems to correlate with the number of mesothelial cells in the effluent, could indicate severe mesothelial cell damage [32]. Further investigations confirmed these results and added new potential biomarkers of PM fibrosis [33]. Bruschi et al. focused on the differences in the proteome profile depending on the osmotic agents, suggesting that the use of icodextrin solution removes a greater amount of middle molecule and small protein compared to glucose solution [34]. More recently, the analysis of peritoneal-derived EVs allowed the identification of a large number of vesicle markers. In 2017, Pearson et al. were able to characterize more than 3700 proteins, including some with a known role in peritoneal pathophysiological changes [35]. Considering the growing interest in this research field, here, focusing on data available in pediatric populations, we reviewed the principal evidence exploring the potentiality of the proteomic analysis of PDE and EVs as a source of novel biomarkers in pediatric peritoneal dialysis.

## 2. Proteomics in Pediatric Peritoneal Dialysis

The proteome is the entire set of proteins expressed in a defined biological sample. Protein analysis of complex samples is conducted with different combined techniques like mass spectrometry (MS) [42], two-dimensional electrophoresis (2-DE), liquid chromatography, microarrays, or protein chips. The development of these tools led to an exponential increase in data volume and complexity to be understood and interpreted. Proteomic analysis of PDE and PM provides a better understanding of biological processes and could be essential to obtain information about specific PM phenotypes, dialysis adequacy, and PD-related complications, such as peritonitis and PM failure until the extreme picture of EPS. Moreover, it may be of help in elucidating the mechanisms of EMT, peritoneal membrane remodeling, and fibrosis [19]. In 2007, Sritippayawan et al. published an interesting study about the proteome profile of PDE obtained from patients with peritoneal membranes in different states of integrity and activity and defined the transport and elimination rate of different small molecules. They classified peritoneal membranes with the use of the peritoneal equilibration test (PET) as high (H), high average (HA), low average (LA), and low (L) transporters. This analysis was conducted on 20 patients, 5 for each peritoneal state of transport, and revealed a significant difference among groups in the levels of five proteins contained in PDE. The first one is serum albumin, lower in H and HA transporters than in LA and L ones, then α1-Antitrypsin, complement component C4A, immunoglobulin κ light chain, and apolipoprotein A-I that, in reverse, are lower in L and LA transporters if compared with the other groups. Immunoglobulin κ light chain level also tended to be higher in patients who had had peritonitis [43]. Regarding the influence of previous peritonitis on the proteomic profile of PDE, Lin et al. conducted an interesting study published in 2008. They selected 16 patients on peritoneal dialysis and performed a mass spectrometric analysis of their PDE samples [44]. Thus, they identified β2-microglobulin (B2M) as a biomarker associated with PD peritonitis, using an unbiased protein profiling. These data confirmed what was previously described by Carozzi et al., who showed that B2M, similarly to interleukin-1 (IL-1) and leukotriene B4 (LTB4) concentrations, were elevated in CAPD patients with bacterial peritonitis [45,46]. B2M was also studied as a PM injury marker by Minami et al. in 2007, who observed that B2M was significantly higher in patients treated with icodextrin-based peritoneal dialysis solution (ICO) when compared to glucose-based solution. Moreover, a positive correlation was also found with hyaluronic acid, interleukin-6, and matrix metalloproteinase-2, considered markers of inflammation and peritoneal damage, suggesting the suboptimal biocompatibility of ICO solutions [41].

In the same way, Zavvos et al. in 2017, examined PDE by searching for potential biomarkers to predict or confirm EPS, intending to stratify at-risk patients and make an earlier diagnosis [47]. They performed a prospective study in well-characterized populations and proved that, compared with patients with stable membrane function, in EPS patients, some proteins are differentially expressed. Therefore, intelectin-1, dermatopontin, gelsolin, and retinol-binding protein-4 levels were higher in patients with EPS than in ones that had just commenced peritoneal dialysis. In contrast, apolipoprotein A-IV and a1-antitrypsin, both presenting anti-inflammatory properties, were significantly lower in EPS patients. More interestingly, some molecules, such as collagen-α1(I), γ-actin, and Complement factors B resulted high up to five years before the development of EPS. These findings showed the potentiality of proteomic analysis not only for the description of disease features but also as a predictive tool for PD complications. To better understand the mechanisms involved in fibrosis, Strippoli et al. provided proteomic analysis of mesothelial derived cells upon mechanical stretching, revealing increased collagen-related proteins and many others involved in the TGF-β pathway, contextually with a reduction of epithelial markers [48]. These data corroborated the evidence of the interplay role between TGF-β signaling and EMT in peritoneal fibrosis [49]. Finally, an interesting study by Ferrantelli et al. investigated the role of N-glycosylation in PDE proteins [50]. They found that specific glycosylation traits significantly correlate to D/P creatinine at PET and that the same traits were associated with TGF-β1 and VEGF. However, despite the increasing number of proteomics studies on the adult dialysis patient population, it is not yet possible to identify a statistically robust clinical biomarker [51]. Parallel to this area and in the light of the presenting background, pediatric studies have been developed. The proteomic composition of the PD fluid in children has been the object of study for several years by different groups. Raaijmakers et al. in 2008, collected dialysate fluids from nine pediatric patients giving a first representative overview of the PDE proteome. The majority of the proteins were shared among all patients and belonged to the extracellular matrix, reflecting the clear relation of PD fluid with the extracellular space. They identified several frequently occurring proteins, like acute phase proteins, complement factors, hormones, coagulation factors, and apolipoproteins. In addition, they also characterized some interesting new proteins like gelsolin, which has a protective role in mesothelial cell damage and against infections, intelectin-1, with a possible role in the defense against intestinal bacterial permeation and parasites, and paraoxonase, committed to protecting lipoproteins from toxic oxidative modifications [40]. Another study published in 2015 by Bruschi et al. found a mean of 700 new proteins recognized in the PDE of 19 pediatric patients [39]. They used an integrated approach of 2-DE and Combinatorial Peptide Ligand Library technology to enhance the capture of undetected proteins overcoming the problem of low abundance proteins masked by high abundance ones. In the range from 1 to 38 months covered by the study, they identified 29 potential biomarkers spots changing over time that could be potentially useful to identify patients with subclinical inflammation and/or developing peritoneal membrane fibrosis. Furthermore, considering the TGF-b signaling cascade and the activation of the complement system, in 2017, Bartosova et al. conducted a transcriptomic and proteomic analysis of peritoneal samples on a selected cohort of children with ESRD before dialysis and upon starting chronic PD [38]. They specifically investigated the PD-induced microvasculature damage mechanisms of the peritoneum vessels previously isolated by microdissection. They demonstrated, in the PD parietal arterioles, marked activation of C1q and terminal complement complex, which correlated with the level of intraperitoneal glucose exposure, the abundance of phosphorylated SMAD2/3, and degree of vasculopathy [38]. Subsequently, in 2021 the same authors performed a multi-omics study comparing omental and parietal peritoneal tissues in children before dialysis and on chronic PD with fluids containing very low or high GDP concentrations. They performed a cross-omics analysis of RNA and proteins with strong overlap between transcriptomic and proteomic. In the vessels of high GDP treated patients, they registered more lumen narrowing with an increase in the intima thickness, while the protein quantification verified increased proapoptotic activity, cytoskeleton disintegration, and immune response. Omics analysis also evidenced an inverse correlation between arteriolar endothelial cell counts and pSMAD2-3 induced TGF-β and IL-6. These findings question the use of high-GDP PD fluids, considering the significant cardiovascular risk of CKD patients and the suspected impact of GDP in accelerating vasculopathy development, and suggest focusing research on improvement of GDP clearance or safeguarding vascular endothelial integrity in PD patients [37].

## 3. Proteomics Applied to Extracellular Vesicles and Exosomes

**Figure 2:**
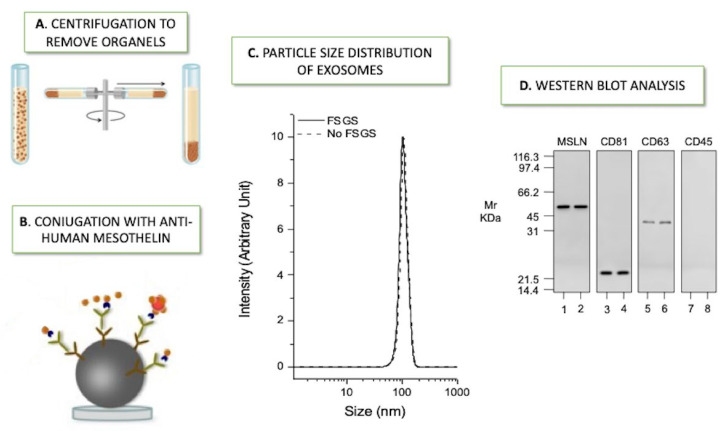
Isolation and characterization of mesothelial exosomes. Steps of the isolation and characterization of mesothelial exosome from peritoneal dialysis effluent: (**A**) Aliquots underwent a series of centrifugation cycles to remove mitochondria, other organelles, and microvesicles to obtain a purified fraction of exosomes. (**B**) Enriched exosomes fractions were mixed with polyclonal biotin-conjugated anti-human mesothelin. (**C**) Exosome size was determined by Dynamic Light Scattering, revealing a Gaussian distribution profile with a typical mean peak at 100 ± 5 nm (**D**) Western blot analysis revealed that the exosomes of both groups were positive for mesothelin (MSLN), CD81, and CD63, but not for CD45 (FSGS = focal segmental glomerular sclerosis).

As stated above, PDE represents an important basin of potential clinical biomarkers that could be useful for foreseeing individual risk of evolving PM fibrosis. It is well-known that cells communicate information via the secretion of soluble elements or by direct interaction. Furthermore, all cell types discharge membrane-derived vesicles that can exert their effect on both surrounding and distant cells [52]. According to the updated guidelines of the International Society for Extracellular Vesicles of 2018 (MISEV2018), these membrane-derived vesicles, known as EVs, are identified as lipid-bound nanoparticles not able to replicate, released by cells, and are present in all bodily fluids [53]. The EVs contain various molecules, such as miRNAs, mRNAs, proteins, and lipids involved in cell-to-cell communication and several other physiological and pathological mechanisms [54]. After release into the extracellular space, the EVs reach their target and release their cargo information. The uptake of EVs appears dependent on the recipient cell and may require specific receptors or involve direct plasma membrane fusion, phagocytosis, or endocytosis. MISEV2018 categorized EVs into three groups by their biogenesis: exosomes, microvesicles, and apoptotic bodies. Exosomes, ranging from 30 to 120 nm, are generated by endosomal pathways, while microvesicles (100–1000 nm) are produced by budding from the cell membrane [53]; both extracellular vesicle categories enclose cytoplasmic and membrane proteins and RNAs, however exosomes typically provide some other constituents, such as receptors and major histocompatibility complex (MHC) molecule [54,55]. The apoptotic bodies are 800–5000 nm sized particles discharged from dying cells and include only nuclear fractions and cell organelles [56]. The choice of the proper method for the isolation and analysis of EVs is challenging. There is still a lack of standard procedures for each biological fluid and/or clinical application. The current isolation techniques rely either on size and density differences between EVs, such as ultracentrifugation, precipitation, filtration, chromatography, or immunoaffinity-based approaches using specific antibodies to capture the corresponding proteins expressed on the surface of EVs. Routinely, researchers combine more than one technique. Previous reports have found EVs in the PDE that formed and got together in the peritoneal cavity throughout PD, perhaps as a stress reaction induced by PD solutions. A specific focus on mesothelial-derived EVs has been done in this clinical setting which represents a major source of EVs in PDE. Indeed, mesothelial injury is crucial in PM failure in response to chronic exposure to dialysis solutions [57,58]. Proteomics is capable of examining EVs in inflammatory diseases, mostly to detect alterations in the expression of proteins in normal against pathologic conditions, which allows the identification of new biomarkers and elucidation of the underlying processes in inflammatory diseases strictly dependent on their cell origin, leading to a protective or pathological effect [59]. In this view, a rapid recognition and risk assessment of PM failure in PD patients is an important goal for the nephrologist. Carreras-Planella et al. outlined, through a prospective study, that changes in proteome content of EVs originated from PDE (PDE-EVs) anticipated the modification of PET in a small cohort of PD patients [60]. Specifically, they found an increase in endoglin expression over time in the stable PM function group compared to the unstable one. Endoglin is part of the TGF-β receptor complex, previously associated with anti-inflammatory effects [61]. Other interesting biomarkers evidenced were mesothelin and THY-1, major players in cell-to-cell and cell-matrix interaction. Fang et al. tried to evaluate the expressed components in PDE-EVs from PD patients with distinct peritoneal solute transport rate (PSTR) based on a PET test and subsequently recognized key proteins involved in pathways of the inflammatory system. Sixty patients on PD were divided into high/high average transport group (H/A) and low/low average transport group (L/A). The ability of proteomics to analyze expressed proteins and potential biomarkers in PDE-EVs allowed the characterization of a greater expression of glycoprotein 96 (GP96) in PDE-EVs of patients with high PSTR. GP96 belongs to the family of chaperons and participates in the signal of integrins and toll-like receptors (TLRs), playing a central role in innate and adaptative immunity. Successively, the researchers validated their results in vitro, showing that GP96 increased the secretion of proinflammatory cytokines (IL-1β, IL-6, TNF-α, and IL-18) in peritoneal derived macrophages and in a PD rat model where the GP96 inhibition reduced peritoneal inflammation response through the reduction of inflammatory cells, cytokines, and chemokines (CCL2, CXCL1, and CXCL2). Their findings suggest that GP96 can be a potential indicator of peritoneal inflammation and PSTR in PD patients [62]. In the latest years, among the other extracellular vesicles, the analysis of exosomes has earned the greatest interest in the field of new diagnostic and therapeutic possibilities [63]. Exosomes exert pleiotropic actions, including the delivering effectors as a cargo, regulating immune responses, and playing a pivotal role in intercellular communication. This fundamental property was reported in the Wen et al. study, in which exosomes pulled out from high glucose-treated renal tubular stimulated the shift of fibroblasts into myofibroblasts in renal tissue, implying that exosomes play a role in the communication between the two cell types under pathological conditions [64]. In renal tissue, exosomes are released from all the cells of the nephron and the urogenital tract and, in major part, by the epithelium or podocytes [65,66]. Given their essential role in managing biological processes, it is not surprising that proteomic analysis of exosomes may represent an important tool to study PM and increase PD efficiency. Corciulo et al. investigated the expression of the water channel Aquaporin 1 (AQP1) in mesothelial cells. Subsequently, they observed that AQP1 is released in the PDE through exosomes and highlighted a positive correlation between the amount of AQP1, ultrafiltration, and solute transport through PM, classical parameters used to outline PD adequacy [67]. Given the large-scale interest in exosomes as a source of potential biomarkers, several biomedical research has been focused on evaluating the potential role of these nanoparticles in the future of clinical practice. In 2021, Bruschi et al. examined PDE by assessing the protein content of mesothelial exosomes in PD pediatric patients with focal segmental glomerular sclerosis (FSGS) compared to no FSGS patients [36]. The purpose of the research was to understand if the first are more susceptible to developing PM fibrosis since FSGS is a significant cause of steroid-resistant nephrotic syndrome in the pediatric age leading to ESKD. They used a purified fraction of PDE exosomes to avoid the risk of masking important biomarkers by high molecular weight proteins. Afterward, a specific subset of exosomes was selected using a biotinylated anti-human mesothelin antibody and streptavidin magnetic beads (Figure 2), identifying 2490 proteins, 40% involved in fibrosis, most of them being part of the TGF-β signaling pathway. Tissue inhibitor matrix metalloproteinase 1 (TIMP1) was a significant down-regulated protein in FSGS, while Annexin A13 (ANXA 13) resulted as the most promising up-regulated potential biomarker to distinguish peritoneal dialysis effluent exosomes of FSGS patients from no FSGS ones. ANX family has been investigated in oncology, and it seems to be involved in numerous intracellular and extracellular pathways, such as inflammation, coagulation, and fibrinolysis [68]. A recent study by Xue et al. showed that many EMT mediators are modulated by Annexin A13 and that its overexpression could accelerate EMT activation, increasing the proliferation and migration of lung adenocarcinoma [69]. The detailed research studies about exosomes of the latest years provided new insights also in the direction of fixed tissue. The isolation of these nano-vesicles in tissues may be important in understanding their molecular mechanism in healthy and pathological microenvironments. In 2019, Gupta et al. accomplished the goal of isolating bovine vitreous humor EVs and quantifying their concentration using nanoparticle-tracking analysis. In particular, they figured out that standard formalin fixation induced the decline of Evs from tissue, whereas using 1-ethyl-3-(3-dimethylaminopropyl) carbodiimide holds Evs in situ [70]. In 2020, Hurwitz et al. published a detailed protocol for obtaining small Evs of interest from an entire fresh or frozen tissue (including brain and tumor specimens). Their protocol seemed to help define functions of exosomes of different sizes, providing new perspectives for further ex vivo characterizations of vesicles with biomedical significance, such as expressing the disease progression in a wide spectrum of disorders [71]. Furthermore, to delineate the biological function and therapeutic potentiality of exosomes in the peritoneal dialysis branch, standardized protocols are still needed.

Figure 2, part C, and part D were reproduced and modified under the terms of the Creative Commons Attribution 4.0 License. The Bruschi et al. Proteomic profile of mesothelial exosomes isolated from peritoneal dialysis effluent of children with focal segmental glomerulosclerosis. Scientific reports 2021, 11, 20807.

## 4. Conclusions

Technological improvement of the last decades has led to new horizons thanks to the availability of new scientific resources. Proteomics and machine learning approaches, such as the ability to isolate and analyze EVs, are enhancing our knowledge in bio-molecular science in a stunning manner. In particular, the application of proteomic analysis to EVs allows the identification of a high number of biomarkers and helps us in discovering hidden physiological and pathological mechanisms (Table 2). In PD, many mechanisms affecting the PM function are still unclear, and very few strategies are available to counteract PM loss of function and EPS. This could be particularly important in children because of the potential long lifetime exposition to dialysis. The studies published until now have broadened our horizons on many mechanisms involved in PM pathology, including fibrosis, EMT, sclerosis, and neo-angiogenesis. Indeed, PDE is an optimal and non-invasive source of samples of EVs for proteomic analysis. Nevertheless, proteomic analysis and studies regarding EVs are too little in PD, especially in the pediatric setting, and most of them are limited by the retrospective design, the small patient cohorts, and the absence of the evaluation of the impact of residual renal function.

On the other hand, many issues stay still open and could be the objects of future studies on the topic. So, all the molecules proposed as biomarkers should be validated in larger populations, also to evaluate their correlations with hard clinical outcomes, such as PD failure and EPS development. Similarly, the possibility of translating the knowledge about the new biomarkers into effective tools to guide the clinical management of PD patients in the context of precision medicine remains to explore.

Certainly, further improvements in technology with the development of less costly devices, shorter analysis time, and the increased maximum number of processing samples will probably encourage more prospective studies with larger populations. These will lead us to a step forward in the knowledge of biomolecular mechanisms of PM and our approach and strategy to PD pathology and complications in pediatrics.

## Figures and Tables

**Table 1:** Potential peritoneal-derived biomarkers in pediatric PD patients.

Biomarkers	Main Findings	Reference
ANXA13	Most significant potential biomarker in detecting peritoneal dialysis effluent exosomes of FSGS from No FSGS patients	[36]
TIMP1	Down-regulated protein in FSGS	[36]
PTP4A1	PD vintage and decreased PM function	[36]
CENP-E, FCN2	Up-regulated proteins in FSGS	[36]
Caspase-3, IL-6, ZO-1	Lumen narrowing of parietal peritoneal arterioles of patients exposed to high-GDP	[37]
C1q and C3d	Abundance in PD-associated glucose exposure; correlation with the degree of arteriolopathy and high level of p-SMAD2/3	[38]
p-SMAD2/3	Microvasculature damage mechanisms of the peritoneum vessels	[38]
Intelectin-1	Inflammation and fibrosis	[39]
	Defensive role against intestinal bacterial permeation and against a parasite	[40]
Cystatin C and B2M	Peritoneal dialysis efficiency	[41]
Paraoxonase	Protection against toxic oxidative modification; possible correlation with early atherosclerotic changes in peritoneal dialysis	[40]
Gelsolin	Protective role in mesothelial cell damage against infection	[40]

Abbreviations: Annexin 13 (ANXA13), Inhibitor matrix metalloproteinase 1 (TIMP1), Focal segmental glomerulosclerosis (FSGS), peritoneal membrane (PM), PTP4A1 (Protein Tyrosine Phosphatase 4A1), Ficolin-2 (FCN2), Centromere-associated protein E (CENP-E), interleukin-6 (IL-6), peritoneal dialysis (PD), glucose degradation products (GDP), Phosphorylated-suppressor of mothers against decapentaplegic 2/3 (p-SMAD2/3), Beta2-microglobulin (B2M).

**Table 2:** Studies published on proteomic analysis in PD in children and proteomic analysis on PDE-derived EVs.

Study	Population/Patients and Study Design	Main Topics/Research Aim	Main Biomarkers	Proteomic Analysis	AI Methods and Approaches	Ref.
Fang J. et al. 2022	ADULTS Sixty patients undergoing peritoneal dialysis (PD) divided into two groups: high/high average transport group and low/low average transport group	Proteomic analysis on PDE-EVs to identify potential biomarkers related to different Peritoneal membrane phenotypes	Glycoprotein 96 (GP96)	Liquid chromatography-tandem mass spectrometry. Mass spectrometer (Q Exactive HFX) coupled with nanoLC1200 system	Spectronaut 12.0 Pulsar (Biognosys). Data-dependent acquisition) technology. Blast2Go software was applied to associate Gene Ontology (GO) terms with the differentially expressed proteins	[62]
Bruschi M. et al. 2021	PEDIATRIC 6 patients with FSGS vs. 6 pediatric patients affected by other primary renal diseases (no FSGS)	Comparative proteomic analysis of mesothelial exosomes from PDE	Annexin A13 (ANXA13), Centromere-associated protein E (CENP-E) Ficolin-2 (FNC2), inhibitor matrix metalloproteinase 1 (TIMP1) Protein Tyrosine Phosphatase 4A1 (PTP4A1)	Orbitrap Fusion Tribrid mass spectrometer (ThermoScientific)	Andromeda engine, incorporated into MaxQuant software, was used to search spectra against Uniprot human database. Weight gene co-expression network analysis (WGCNA) package in R. T-test, machine learning methods such as nonlinear support vector machine (SVM) learning, and partial least squares discriminant analysis (PLS-DA)	[36]
Bartosova M. et al. 2021	PEDIATRIC 107 CKD5 patients and 90 patients on PD with PD fluids containing very low or high concentrations of GDP.	Impact of GDP on vasculopathy of children in chronic peritoneal dialysis. Microdissected arterioles were isolated for transcriptome and proteome analysis (*n* = 8 in CKD5, *n* = 6 for high-GDP, and *n* = 5 for low-GDP group)	Caspase-3, TGF-β-induced-pSMAD2/3 interleukin-6, zonula occludens-1 (ZO-1)	Liquid chromatography–mass spectrometry (QExactive. Thermo Fisher)	Data were submitted to PRIDE (Proteomics Identification Database). Ingenuity Pathway Analysis software (Qiagen, Hilden, Germany). Similarity data (edges representing shared genes) were generated using R and visualized using Cytoscape 3.8.0	[37]
Carreras Planella L. et al. 2019	ADULTS 11 patients. Follow-up 24 months, collecting samples every six months.	To outline the peritoneal dialysis-efflux—extravesicles (PDE-EV) proteome capacity of showing alterations much earlier than PET)	Endoglin, Thy-1 membrane glycoprotein (THY-1 or CD90) and biglycan (BGN), kininogen-1 (KNG1)	Liquid chromatography–mass spectrometry (LC-MS) (VelosOrbitrap-Thermo Fisher Scientific， Carlsbad, CA, USA)	Data were analyzed using Progenesis QI for proteomics software v3.0 (Nonlinear dynamics, Newcastle upon Tyne, UK). Peak lists generated s were analyzed with the Mascot search engine (v2.2, Matrix Science, London, UK). Protein identification was performed using the SwissProt-human database. Protein enrichment analysis was performed using Gene set enrichment analysis software (GSEA v3.0, Broad Institute, Cambridge, MA， USA) and GSEA Molecular Signatures Database (MSigDB v6.2, Broad Institute, Cambridge, MA, USA)	[60]
Bartosova M. et al. 2018	PEDIATRIC Peritoneal arterioles were obtained from patients with CKD5 (*n* = 15), established PD (*n* = 15), and healthy control (*n* = 5)	Multi-omic analysis to understand the mechanisms of CKD-associated arteriopathy. They showed activation of the arteriolar complement system and correlation with severity of arteriolar vasculopathy in PD	C1q and C3d (terminal complement complex), pSMAD2/3	LC-MS. Q Exactive mass spectrometer (Thermo Fisher Scientific, Carlsbad, CA, USA)	Data were processed and searched against the human SwissProt database with Andromeda search engine using MaxQuant. Gene enrichment analyses were conducted using the PANTHER online database.	[38]
Pearson LJ. et al. 2017	ADULTS 8 patients on stable PD	To demonstrate EVs in PDE and to characterize the related proteome	Mesothelin and cancer cell antigen 125 (MUC16). vWF, CD109, CD14 and its coactivator lipopolysaccharide binding protein. EMT-related proteins: E-cadherin and extracellular matrix proteins collagen I and III, respectively. αSMA, TGF-β related proteins.	LC-MS. mass spectrometer (Q Exactive Plus Hybrid Quadrupole-Orbitrap, Thermo Fisher Scientific, Carlsbad, CA, USA) through an EASY-Spray nanoelectrospray ion source (Thermo Fisher Scientific).	Raw data were searched by X! Tandem (CYCLONE, 2013.2.01) against human databases (ENSEMBL v.76 Homo sapiens GRCh38). Gene ontology was performed using David bioinformatics resource 6.8. Protein lists generated were compared using gene ontology enrichment analysis and visualization tool, GOrilla	[35]
Carreras-Planella L. et al. 2017	ADULTS 9 patients in PD divided in two groups: Newly-enrolled Patients and Longer-treated Patients	Identifying, isolating, and characterizing peritoneal dialysis efflux-extravesicles of patients on PD	CD9 CD63- CD81 Galectin 3-binding protein (LGALS3BP) Ezrin (EZR)	LC-MS/MS on a LTQ Orbitrap Velos (Thermo Fisher, Carlsbad, CA, USA).	Data were analyzed with Max Quant software against Uniprot human database. Further analyses were made using the Intensity-Based Absolute Quantification (iBAQ) values obtained from MaxQuant, and analyzed using Perseus software (version 1.5.6.0), InteractiVenn, and the EVs specific databases EVpedia, Exocarta, and Vesiclepedia. Subsequent analysis was performed Non-supervised hierarchical clustering approach	[72]
Bruschi M. et al. 2015	PEDIATRIC 19 patients with different kidney disease (primarily kidney dysplasia in 6 cases, nephronophthisis in 5 cases).	Proteomic characterization of PDE samples collected in patients with different APD treatment by the combined use of Combinatorial Peptide Ligand Library (CPLL) technology and two-dimensional electrophoresis	Gelsolin, intelectin-1	Matrix-assisted laser-desorption ionization (MALDI)—Mass Spectrometry analy- sis.	PDQuest Advance software for 2-DE experiments and QuantyOne software (Bio-Rad, Hercules, CA, USA) for western blot experiments.	[39]
Bruschi M. et al. 2011	PEDIATRIC 16 patients with different kidney diseases	Proteome profile of PDE obtained with icodextrin or glucose-based solutions	β2-microglobulin cystatin C leptin.	LTQ linear ion trap mass spectrometer (Thermo Electron, San Jose, CA, USA) coupled to an HPLC Surveyor (Thermo Electron)	Protein identification was performed using SEQUEST software (Thermo Electron, San Jose, CA, USA) All digitalized images were analyzed with PDQuest Advance or QuantyOne software (Bio-Rad, Hercules, CA, USA)..	[34]
Raaijmakers R. et al. 2008	PEDIATRIC 9 patients in PD	To obtain the first representative overview of the proteome of PDE. Identified proteins in PDE reflect local peritoneal processes	gelsolin intelectin paraoxonase	cyclotron resonance mass spectrometer (LIT FT-ICR MS)	Data were searched against the NCBI database using the Mascot search program. Protein identifications were validated and clustered using the PROVALT algorithm. Gene ontology classifications were made with Protein Center (www.proxeon.com, accessed on 1 April 2022). To provide an estimation of protein concentration exponentially modified protein abundance index (emPAI) was used	[40]

Abbreviations: Artificial intelligence (AI); Peritoneal dialysis (PD); Automatic PD (APD), Peritoneal dialysis effluent (PDE); extracellular vesicles (EV); glucose degradation products (GDP); peritoneal equilibration test (PET); focal segmental glomerulosclerosis (FSGS): chronic kidney disease (CKD).

## Data Availability

Data supporting the findings of this study are available from the corresponding author on request.

## Abbreviation

Peritoneal dialysis (PD), end-stage kidney disease (ESKD), peritoneal membrane (PM), mesothelial cells (MC), hemodialysis (HD), chronic kidney disease (CKD), ultrafiltration failure (UFF), encapsulating peritoneal sclerosis (EPS), high-glucose degradation products (GDP), transforming growth factor (TGF)-β1, epithelial-mesenchymal transition (EMT), peritoneal dialysis effluent (PDE), alpha-SMA (alpha-smooth muscle actin), mass spectrometry (MS), two-dimensional electrophoresis (2-DE), peritoneal equilibration test (PET), high transporters (H), high average (HA), low average (LA), and low transporters (L), β2-microglobulin (B2M), interleukin-1 (IL-1), leukotriene B4 (LTB4), icodextrin (ICO), the International Society for Extracellular Vesicles of 2018 (MISEV2018), extracellular vesicles (EVs), major histocompatibility complex (MHC), proteome content of EVs originated from PDE (PDE-EVs), peritoneal solute transport rate (PSTR, transport group (H/A), low/low-average transport group (L/A), glycoprotein 96 (GP96), toll-like receptors (TLRs), aquaporin 1 (AQP1), focal segmental glomerular sclerosis (FSGS), inhibitor matrix metalloproteinase 1 (TIMP1), annexin A13 (ANXA 13), 1-ethyl-3-(3-dimethylaminopropyl), mesothelin (MSLN), mass spectrometer (Q Exactive HFX), gene Ontology (GO, centromere-associated protein E (CENP-E), ficolin-2 (FNC2), protein Tyrosine Phosphatase 4A1 (TP4A1), weight gene co-expression network analysis (WGCNA), support vector machine (SVM), partial least squares discriminant analysis (PLS-DA), zonula occludens-1 (ZO-1), PRIDE (Proteomics Identification Database), biglycan (BGN), kininogen-1 (KNG1), gene set enrichment analysis software (GSEA), GSEA Molecular Signatures Database (MSigDB), mesothelin and cancer cell antigen 125 (MUC16), Galectin 3-binding protein (LGALS3BP), combinatorial Peptide Ligand Library (CPLL), matrix-assisted laser-desorption ionization (MALDI), artificial intelligence (AI)., plasminogen activator inhibitor 1 (PAI-1).
